# Physiology of everyday sleep and physical activity: An exploratory mixed-methods study of multi-sensor wearables for infants and toddlers

**DOI:** 10.3758/s13428-026-02945-x

**Published:** 2026-03-06

**Authors:** Emily Hunter, Niina Kolehmainen, Kianoush Nazarpour, Tim Rapley, Abigail Collins, Christopher Eggett, Craig Williams, Christopher Thornton

**Affiliations:** 1 Newcastle University Population Health Sciences Institute, Newcastle University, Newcastle Upon Tyne, UK; 2 The NIHR Innovation Observatory, Newcastle University, Newcastle Upon Tyne, UK; 3 School of Informatics, The University of Edinburgh, Edinburgh, UK; 4 Department of Social Work, Education and Community Wellbeing, Northumbria University, Newcastle Upon Tyne, UK; 5 Faculty of Health and Life Sciences, Children’s Health and Exercise Research Centre, University of Exeter, Exeter, UK; 6 School of Computing, Engineering, and Digital Technologies, Teesside University, Middlesbrough, UK

**Keywords:** Infants, Children, Parents, Wearables, Wearable technology, Human factors, Behaviour, Sleep, Physical activity

## Abstract

Sleep and physical activity are vital to the health, development, and well-being of young children. To effectively promote these behaviours at the population level, better tools for objectively quantifying them are needed. This hypothesis-generating mixed-methods study explored the potential usability of two wearable sensors to measure physical activity and sleep in young children over multiple days, drawing on physiological measurements. A longitudinal within-case design was employed, in which families with children aged 4–36 months from the North East of England were recruited through playgroups and social networks. Parents and children tested two wearable devices in a structured play setting and at home over a period of 1 week. Data on sleep, movement, and heart rate were collected using the Bittium Faros 180 heart rate monitor and the NAPPA sleep monitoring system. Usability was assessed through researcher observations and parent feedback using ethnographic methods. Wear time, heart rate variability during naps, and ultradian respiration cycles during sleep were analysed. Seven children participated and completed the study. While parents were initially enthusiastic, usability challenges arose. The heart rate monitor was considered uncomfortable, its large size hindered activity, and electrodes were detached by parents and accidently, leading to significant data loss. The NAPPA was easier to use, discreet, and comfortable, but disrupted sleep routines. Additional challenges related to non-parental caregiving resulted in non-wear and/or data loss. These results indicate that wearable devices for young children hold potential but face significant design challenges for longitudinal home use at scale. Co-creation of child-friendly, practical hardware and software is essential for effective, large-scale health monitoring in young children.

## Background

Early-life health behaviours establish fundamental patterns for life-course health and provide potentially powerful opportunities for interventions (Sundell & Angelhoff, 2021). These behaviours shape children’s health identities and lifestyles, as well as physiological and cognitive development and maturation. For example, physical activity at youngest ages influences life-course health (Hidayat et al., 2019), and evidence about the importance of early-life physical activity is uncontested; however, effective interventions to support everyday physical activity and active play for young children are lacking, with substantial gaps in the evidence base (Lum et al., 2022). Similarly, getting enough sleep is now widely accepted as centrally important for children’s cognitive and physical development and maturation (Liu et al., 2022), and disrupted sleep is known to increase long-term risk of ill health (Barrero-Castillero et al., 2022). Children’s sleep problems are one of the most common concerns for parents (Thornton et al., 2023), but evidence about, and research into, children’s daily sleep is limited, and there are no national guidelines or evidence-based best-practice interventions for promoting good-quality sleep.

Wearable technologies are already widely used for promoting health behaviours in adults and older children, and are increasingly recognised as a potential opportunity for population-level health promotion and monitoring of early-life, everyday sleep and physical activity (Matricciani et al., 2019). For example, a collaborative project in the United States recently published a protocol (Pini et al., 2024) to advance the use of wearables in longitudinal research on children’s sleep, activity, and development. However, there is currently a scarcity of published work on the use of contextually embedded digital technologies and methodologies in studying children’s health behaviours as they occur naturally, over time, in daily life (Hu et al., 2024). A population-level, everyday health promotion paradigm is complementary to but also different from the already more advanced use of specific clinical diagnostic devices in young children—for example, monitoring physiology in the context of preventing sudden infant death syndrome (SIDS) (Ul Hasan & Negulescu, 2020), continuously monitoring neonates’ vital signs in critical care settings (Zhou et al., 2024), or assessing neurodevelopment in children at risk of neurodisability (Taylor et al., 2024). In the absence of effective ways to track young children’s everyday activities, there is sparse robust evidence on young children’s everyday health behaviours and how to effectively promote them.

Any new methods, including wearables, must be meaningful and acceptable to young children and parents, as well as being accurate, comfortable, and safe. Collecting data on the everyday health behaviours of children, especially younger children under 36 months, comes with unique challenges. For example, compared to older children, adolescents, and adults, young children have limited responder capacity (cognitive, language, motor); proxy responder data for them often has limited validity, and their data have naturally large within- and between-person heterogeneity and rapid change due to child growth and development. Thus, while studies using adult-oriented wearables and research paradigms are common in adults and teenagers, studies with younger children are significantly limited by these methodological challenges. Existing studies on wearables with children have largely focused on older children, commonly with emphasis on activity tracking (Irvin et al., 2017; Mackintosh et al., 2019; Creaser et al., 2022; Lu et al., 2020). Although consumer devices for infants do exist, for example for measuring sleep, their usability for research remains to be established (De Zambotti et al., 2019). Consequently, young children continue to be overlooked in wearables research, and the related opportunities for life-course prevention remain largely unexplored. While new, large studies with a focus on population health promotion are being established (Pini et al., 2024), it will be some time before these results are reported. Collective, cumulative learning across studies is needed to accelerate learning about the usability of infant wearables for population health research.

In the present study, we explored the feasibility of using of a commercially available Holter electrocardiogram (ECG) monitor and a respiration and sleeping position monitor (Ranta et al., 2021) in children aged 4–36 months over several days at home. We sought to investigate how these wearables are received and responded to by children and their parents, and to what extent the wearables can be used to collect high-quality data on children’s everyday health behaviours, with the families’ everyday lives, activities, and experiences as a central focus.

## Methods

This was an exploratory study using a longitudinal, multiple-case, mixed-methods observational approach. The design was guided by a set of predefined key questions (see Supplementary Section 3) covering the feasibility and acceptability from the child and parent perspective as well as for collecting high-quality data. The study focused on generating multidimensional descriptions through diverse data, but not hypothesis-testing. The full protocol (Thornton et al., 2022) approved by the Newcastle University Research Ethics Committee (Ref. 2408/24634) has been published online. All supplementary files can be accessed by the online link provided in the *Supplemental Files* section.

### Recruitment

We sought to recruit 10–12 children aged 4–36 months and, for each child, at least one adult offering everyday parenting care for them (from here on, referred to as “parent”). There were no exclusion criteria for the children’s developmental state, capacity, or health except for a strong rationale to consider that either device used for data collection could negatively affected the child’s health (e.g. allergies to the materials used).

Recruitment took place in the North East of England, UK. Pre-prepared materials inviting families with young children to join the study were disseminated as follows: in person at three community-based playgroups; an online advert on a North East Parents social media site; and an email invitation circulated via a workplace mailing list for a mix of office-based companies, public sector research centres, and university staff.

In line with the purpose and focus of the study, it was not powered for quantitative testing of hypotheses. With seven families successfully recruited, an a priori precision calculation indicated that the proportion of usable days or nights of data could be estimated with 19% precision. We therefore emphasise that, while this is adequate for feasibility or pilot study goals, it is not sufficient for detecting small differences between groups or for generalising to larger populations. We also emphasise that external validity is limited because the sample is unlikely to be representative of the overall heterogeneity of the population (see Supplementary Section 4).

### Measures

**Fig. 1:**
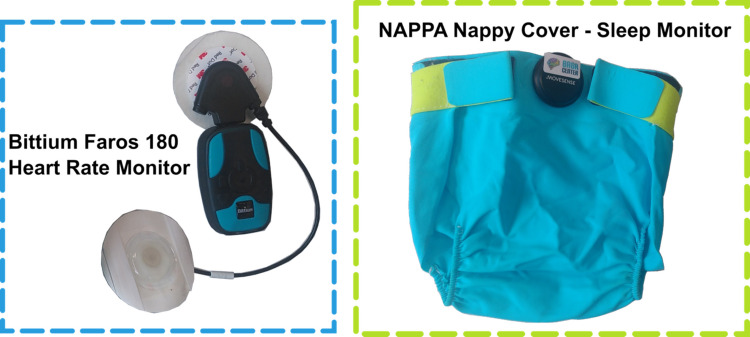
Wearable sensors used in this study. Left: Bittium Faros 180 with paediatric electrodes and a short-cable two-electrode configuration. Right: NAPPA sleep respiration and movement monitor as nappy cover

Data were collected on child behaviours and physiology as well as the feasibility and acceptability of measurement using wearables (Fig. 1), observations, ethnographic methods, and questionnaires (Supplementary Table 1). In children, accelerometery is typically used in studies that seek to objectively quantify sleep (Armstrong et al., 2019) or physical activity (Kolehmainen et al., 2023). Accelerometers are easy to use and acceptable for parents and even for younger children. They typically measure linear acceleration in multiple planes, and in this way can provide insights into how much the child is moving while on their bed and how much the child’s body is moving on the different sagittal axes while awake. Accelerometers lack the ability to provide insight into sleep quality, or to separate the child movements from parent movements (e.g. child being carried or pushed on a swing). Physiological measures, such as heart rate and respiration rate, obtained directly from the child provide a potential avenue for richer data about these health behaviours. However, there are currently no commercial solutions or protocols that tap into robust physiological signals from the child, at large scale, with easy data collection for parents and efficient analysis, ideally in real time, reflective of children’s natural environments and experiences.

Two wearable technologies were selected for the study, with emphasis on accessible, low-cost, and open data options, and on maximising child and parent choice. The Bittium Faros 180, referred to as a heart rate monitor, was selected to collect data on physical activity, heart rate, and heart rate variability. It records an ECG and accelerometery and attaches to the body using two recording electrodes. Parents were not required to interact with the device, other than to remove or attach it. Further details on the use of the Bittium Faros 180 are provided in Supplementary Section 2. The NAPPA sleep monitoring system (de Sena et al., 2024), with napping pants, was selected to collect data on position, movement, and respiration during sleep. It is a wearable accelerometer and gyrometer clipped to a nappy cover, that can be worn by an infant or child. It measures their respiration rate and sleeping position. To operate the NAPPA, a mobile phone app must be used to begin recording and to log the data once recording is complete. Parents were required to set the device to record once their child was wearing it in the evening; in the morning they were required to stop the recording and log the data to the phone to record the night's sleep data. Other devices considered are presented in Supplementary Section 1.

Observations of parent and child responses to the two wearables were made throughout the study. We noted any reactions, views, and questions, focusing on responses in terms of acceptability and feasibility. Some of the observations were also recorded with audio and video. Parent diaries, where parents and children collected their own data detailing their experiences, were offered as an optional way for children and parents to contribute their perspectives to the study. These were supplemented by an ethnographer-researcher visiting the child and family at home, to observe and discuss their experience with the wearables and collect feedback through conversations and unstructured interviews during activity within the child’s everyday context.

The data collection consisted of four sequential stages, each involving a quantitative and qualitative component (Table 1). The transition between the stages was offered as an explicit opportunity for the parent and child to exit the study. Throughout, we emphasised the exploratory, playful nature of the study to the children and parents. We stressed that negative views and critique provided us insights into how the wearables could be improved and as such were at least as valuable as any positive views. We emphasised that children and parents could withdraw at any time without being asked for a reason and regularly gave them opportunities to do so.

**Table 1:** The four stages of piloting and data collection in the study

Stage 1: Introduction and device setup at the lab: “Play Test Session”	In-person play session at a structured setting (a baby lab), objectives to • Meet and build rapport with the child and parent(s) • Explain study in detail and, if agreed and consented to, fit the heart rate monitor • Collect initial data on feasibility and acceptability through wearable testing and observations
Stage 2: Video-recorded structured play session at the lab	A 10–20-min video recording taken in the session with the wearable being worn, designed to • Record the child engaging in purposive activities of increasing physical intensity (categorised into no/minimal movement, light activity, intense activity) • Record and observe initial device wear and identify any initial issues encountered
Stage 3: Multi-day physiological and ethnographic recordings at the participant's home	The multi-day, “take-home” element of the study for • Child and parent(s) to take the wearables home to trial on their own • Establishing whether the devices were acceptable and usable • Parent(s) to record their daily activity and experiences in diary provided by study
Stage 4: An ethnographic home visit	A visit from a researcher during the multi-day wear • To observe the child and wearable in real time and in the child’s own context and daily life activities • The researcher listened to the child, the parent, and other people present, and asked questions to better understand them, including discussing their experience with the wearables and collecting feedback

### Analysis

#### Signal quality metrics

For the heart rate monitor data, wear time was assessed using both the ECG and acceleration signal recorded by the monitor, with a detectable heartbeat or persistent movement considered to imply the device was worn. If persistent movement was present within a window without a heartbeat, then the device was considered worn but not correctly attached. The threshold for persistent movement was 13 mg applied to the standard deviation of the vector magnitude of the signal. The signal quality, while the device was worn and attached, was assessed using the number of intervals outside of the physiological range (a threshold of 150 in 10 min), as well as the proportion of the signal for which we could calculate an accurate heart rate and heart rate variability (at least 50 or 100 physiological R–R intervals in the 10-min window, respectively). Full details on how we calculated wear time, heart rate, and heart rate variability are presented in Supplementary Section 4, where we also include details of thresholds on acceleration and ECG signal quality and sensitivity analysis on these thresholds.

For the NAPPA, wear time was assessed by the number of nights worn and the duration of each night’s recording. The quality of the respiration rate signal was assessed using the percentage of signal for which an accurate respiration rate could be calculated. This is determined by a threshold of 0.35 on the autocorrelation of the signal, considered by the developers of NAPPA as an appropriate threshold (Ranta et al., 2021). Average respiration rates were taken from those provided by the NAPPA, and the mean of all values above the autocorrelation threshold were used. Methods used to estimate ultradian sleep cycles from the respiration rate signal are presented in Supplementary Section 4. Across all data from both wearables, processing was performed using Python 3.8.10, and all statistics and signal processing used SciPy version 1.10.1.

#### Calculation of physiological metrics

The ECG measured by the Bittium Faros 180 was processed by software embedded on the Faros device to generate a series of R–R intervals. These R–R intervals were then processed to produce a time series of heart rate and heart rate variability values.

#### Heart rate and heart rate variability calculation

To calculate the heart rate, we took the R–R intervals for each 1-min segment; if there were at least 25 intervals within the physiological range, we considered this sufficient for an accurate heart rate reading for this segment. To calculate heart rate variability, we took the R–R intervals for each 10-min segment, with a minimum requirement of at least 100 intervals per segment. The heart rate variability metric SDRRI (standard deviation of R–R intervals index) was then calculated as the standard deviation of the intervals for each 2-min segment.

The sleep positions and respiration rate were calculated by the NAPPA software. The details of these calculations are described in Supplementary Section 3 of de Sena et al. (2024).

### Reliability of measures

To assess the reliability of measurements taken at home, we calculated the daily/nightly mean of the heart rate, heart rate variability, and respiration rate, along with their 95% confidence intervals and the coefficients of variation. To quantify the difference between devices, we compared the number of successful nights with the NAPPA to the number of successful days with the heart rate monitor. The difference in the proportion of nights and days was calculated for each child, before we applied a Wilcoxon rank sum test on this difference in proportions to assess whether it significantly differed from zero.

Further details on processing methods can be found in Supplementary Section 4, including a table of references to literature supporting our choice of parameters, sensitivity analyses, and details on how we estimate the ultradian cycles in respiration rate.

#### Qualitative analysis

Transcribed videos, as well as notes from the initial play test session, the parent diaries, and the researcher field notes were analysed using content analysis (Krippendorff, 2019). Data were first analysed in relation to each child, allowing for holistic understanding of each case’s experience from start to finish. Later, thematic analysis (Braun & Clarke, 2006) was implemented to cross-analyse experiences, identifying main themes and sub-codes within the accounts to decipher commonalities and contrasts between cases and their experiences.

#### Synthesis

Conceptual, continuous synthesis was undertaken concurrently with analysis of data across sources. This synthesis was informed by ongoing team discussions involving sharing of data and proactive feedback-seeking. Emerging ideas, gaps, and discrepancies in data and emerging description of the phenomenon were noted and further elaborated on, explored, and explained by another set of data. Throughout the study, ongoing, reflective team discussions were held to learn about both the application of the protocol and related research practices. Reflexive diaries were actively used to contribute to knowledge generation, discovery, and quality assurance (Finlay, 2002).

At the end of the study, feasibility was explicitly reflected upon through three criteria: first, the willingness of parents and children to engage with the study, to consent, and to initially use the device; second, whether wearing the device for multiple days could be accommodated into everyday lives by the parents and children; third, whether the devices were able to record sufficient quality and quantity of data to capture the physiology of everyday events.

## Results

From an overall estimated potential participant pool of 2,500 individuals, 10 families meeting the child age criterion approached the team; nine attended an information session, seven consented to data collection, and all seven children (six female, one male, age 15–27 months) and their nine parents were retained for the full study. The non-participation of the two parents who attended the information session but did not participate was due to impracticality of timing related to the study. Five families were recruited via social media and two via the workplace mailing list. In five cases the study involvement was predominantly led by one parent, in one case both parents were involved in all study stages, and in the final two cases participation was primarily led by one parent but both parents participated in the home visit.

From the synthesis, three key clusters of findings emerged: initial impressions, the reality of using the wearables at home, and the potential of the data to describe the physiology of everyday events. The qualitative and quantitative results intersected these, with each section addressing the corresponding feasibility criteria outlined in the methods. *Initial impressions* address the first criterion, about the willingness of parents and children to engage with the study and initially use the device. *The reality of using the wearables at home* addresses the second criterion of whether wearing the device for multiple days could be accommodated into everyday lives. *The potential insight from the recorded data* addresses the third criterion of whether the devices were able to record sufficient quality and quantity of data to capture the physiology of everyday events.

### Initial impressions

The parents, often unprompted, described their intrigue for wearables and the future of wearables as part of their rationale for taking part. This was alongside interests in science and technology, and, most commonly, in wanting to enhance their insights into their child’s daily experiences. Parents discussed these motivations at length, with particular attention given to sleep or sleep difficulties, and wanting to seek more information about this where possible. These stated motivations underpinned parents’ willingness to try out the technologies.

In the play test session, when provided the opportunity to “meet” the wearables, all parents wanted to handle them and try out their features, for example, sticking the electrodes on themselves to test stickiness or touching the fabric. After this exploration and experimentation, all parents stated that they were willing to attempt the first wearable (heart rate monitor) with their child in the play session. Later, all parents chose to take this wearable home to try, as well as the second, also optional, wearable (NAPPA sleep monitoring system).

Across the data, the key indicators of acceptability that were observed were parents and children touching both wearables, trying them on, and commenting positively on the familiar appearance of the NAPPA. Parents also actively encouraged their child to look at, and incited interest in, the wearables, and quickly agreed to take them home for a further trial. The key indicators of unacceptability were verbal or physical hesitation by parents, verbal or physical refusal by the children, in which children preferred to play with other toys rather than engage with the wearable, and children expressing upset when the wearable was moved toward them. Table 2 provides quotes and researcher observations that illustrate children’s and parents’ responses to the initial introduction of the wearables.

**Table 2:** Illustrative responses of children and parents to the two wearables related to the initial introduction and structured play session

Wearable	Play test session Illustrative quotes by parents	Play test session Illustrative observation and video notes
Bittium Faros 180 heart rate monitor	“Let’s try whatever on her, and we’ll see… we’ll see what sticks!” P002^a^ “They [wearables] were much less intrusive than I thought they would be.” P001 “When asked if they wanted to try the device, they would clearly say ‘no’” P005	Child took a particular interest in the Bittium and playing with it. P001 After looking at an electrode, and putting it on their doll, the child let the researcher attach the device. P007 Parent also tries the electrode on their own wrist and states that taking it off is easier than a plaster. P004 Child covered their chest or pushed the Bittium away if someone tried to place it on them, including parent. Continued playing instead, but did stick electrodes on the toys present. P003 Child immediately started crying when their chest was wiped with the wipe (later described as too cold). P006 None of the participants displayed any expressions likening to distress, sadness or anger. P001, P002, P007
NAPPA sleep monitoring system (NAPPA)	“We will be able to get them on when we change her.” P002 Parent quote	Parent thought NAPPA would not be an issue for the child; they wear similar things over nappies while swimming. P006

^a^ P002, Participant ID #

In four of the seven cases, within the structured play session, the monitor was successfully attached to the child and a signal detected (mean recording 17 min). In all of these, the children appeared comfortable, and there were no changes observed in their play and interactions from before to after the wearable having been fitted. For the other three, fitting did not progress due to refusal by the child, expressed by saying “no”, covering their chest with their hands, and/or moving away from the researchers or the wearable. Initial indicators of unacceptability to the children were attributed retrospectively by the parents to unfamiliarity of the wearable and its features, and the novel, or distracting, session environment. No child tried the NAPPA at the play session, as parents expressed preference to do so at home instead. All parents elected to take both wearables home, including those for whom trying the monitor within the session had been unsuccessful, stating that they would like to try in a more familiar environment.

### The reality of using the wearables at home

Five of seven children wore the heart rate monitor beyond the play session; one succeeded in wearing it for the recommended minimum of three full days (Table 3). The two children who did not wear the monitor had refused to do so, and parents attributed this to the monitor’s unfamiliarity.

**Table 3:** Summary of wear time for the Bittium Faros 180 heart rate monitor

Child participant ID	Number of wear times	Total wear time	Wear time attached	Time worn but not fully attached	Number of accidental detachments	Error rate^¥^
P001	2	19 h, 10 min	19 h, 10 min	None	None	1.441
P002	7	4 days, 1 h, 10 min	3 days, 14 h, 20 min	10 h, 50 min	5	0.326
P003^a^	1	6 h, 35 min	0 min	6 h, 35 min	1	NA
P004	5	3 days, 17 h, 20 min	2 days, 13 h, 40 min	1 day, 3 h, 40 min	3	0.491
P005	0	Not worn	Not worn	Not worn	Not worn	NA
P006	0	Not worn	Not worn	Not worn	Not worn	NA
P007	7	1 day, 21 h, 50 min	1 day, 12 h, 10 min	9 h, 40 min	4	1.207

^¥^ Number of R–R intervals outside the physiological range recorded per minute for the time when the device was worn and fully attached

^a^ indicates the heart rate monitor was worn by the child, but no successful heart rate recordings were obtained

Continuous successful wear occurred mostly when parents reported that the child did not notice the monitor, where the monitor could be worn underneath clothes and was not visible to the child. In cases where it was noticed, such as during bathing or changing, children were reported to be surprised and curious about the device, sometimes touching it, but not exhibiting distress or discomfort. Six children wore the NAPPA at home, three of whom succeeded in wearing it for at least three nights (Table 4).

**Table 4:** Summary of wear time for the NAPPA sleep monitoring system

Child participant	Nights	% with autocorrelation > 0.35	Median recording duration (h)
P001	1	35	1 day 09:14:56
P002	3	54	12:47:37
P003	0	0	0
P004	5	44	11:05:17
P005	2	42	16:36:59
P006	2	40	16:37:08
P007	9	49	12:29:25

Table 5 shows the daily/nightly mean heart rate (HR), heart rate variability (HRV), and respiration-derived ultradian period, along with their 95% confidence intervals and coefficients of variation (CV). Mean HR ranged from 120 bpm to 131 bpm, with the CVs indicating that these measures were stable across days for the three children with sufficient data (3–5%). HRV measures were less stable (CV between 10% and 14%), while nightly stability varied among children (3–37%).

**Table 5:** Reliability statistics of three key measures for each child

Subject	HR (mean bpm) [95% CI]	HRV (mean) [95% CI]	Ultradian rhythm period (min) [95% CI]	HR (CV, *n*)	HRV (CV, *n*)	Ultradian rhythm period (CV, *n*)
P001	127 [81, 172]	48 [−14, 110]	80 [NA]	3%, *n* = 2	10%, *n* = 2	NA, *n* = 1
P002	120 [113, 126]	34 [28, 40]	92 [−12, 196]	5%, *n* = 4	12%, *n* = 4	37%, *n* = 3
P003	NA	NA	NA	NA	NA	NA
P004	131 [118, 144]	29 [17, 41]	73 [57, 89]	3%, *n* = 3	14%, *n* = 3	16%, *n* = 5
P005	NA	NA	93 [−28, 213]	NA	NA	10%, *n* = 2
P006	NA	NA	79 [47, 112]	NA	NA	3%, *n* = 2
P007	127 [NA]	32 [NA]	95 [71, 121]	NA, *n*= 1	NA, *n* = 1	32%, *n* = 9

Table 6 shows the number of successful nights or days for each child wearing each device. Three of seven children did not achieve a successful recording with the heart rate monitor at home (although P003 did wear the device, it did not successfully record a heart rate), while one of seven did not wear the NAPPA. We found that while more children wore the NAPPA, there was no statistically significant difference in the proportion of days/nights with successful wear (T = 3, *p* = 0.16).

**Table 6:** Comparing the success of the two devices

Subject	Number of usable NAPPA nights	Proportion NAPPA	Number of usable HR days	Proportion HR
P001	1	0.14	2	0.29
P002	3	0.43	5	0.71
P003	0	0	0	0
P004	5	0.71	3	0.43
P005	2	0.29	0	0
P006	2	0.29	0	0
P007	7	1	1	0.14

* T = 3.0, *p* = 0.16

For the monitor, four of the five children experienced several hours with the device worn but not fully attached, resulting in no heart rate detected (details in Table 3). This was explained by accidental or purposeful detachment, when a parent chose to take a break from wear or discontinued wearable use. Accidental detachment occurred when the monitor became unclipped, caused by the child’s natural movements or from being carried. Sometimes it was clear when this had occurred:

Occasionally it did if she was a bit boisterous, it became unconnected. A couple of times when she’d climb up on the sofa, she’d knock it off. – P004 parent quote.

She’s still carried quite a lot, and every time, if you’re not careful, if you pick her up it pops off. – P007 parent quote.

Other times, detachment would happen inconspicuously, which parents described as hard to identify, only noticing the detachment upon changing, and thus only able to correct this after multiple hours.

Noticed at 13:30 her monitor had become unclipped. It's hard to check because babies/toddlers wear vests so only really get to check when we change her nappy. If it falls off it doesn't fall off completely as it's caught in the vest/nappy so you don't notice. – P002 Parent diary entry.

In cases when the monitor use was purposefully discontinued, this was mostly due to discomfort from the adhesive pads that secured the monitor’s two electrodes. Despite selecting paediatric electrodes for the study, the least adhesive for continuous wear, three cases reported child’s skin reactance to the electrodes, and the adhesive pads sticking firmly, difficult to remove. Parents reported that this caused soreness and pain and stopped wear. Skin challenges featured again, as two children had eczema, and wearing the monitor was difficult in combination with essential skin moisturising emollients. The electrodes could not stick securely and moved around or were removed by the parent to prioritise skincare.

Other feedback that indicated unacceptability in daily contexts were the size of the heart rate monitor, described as too large and bulky, or noticeable or annoying to the child and inhibiting their movement or catching on clothing (Table 7). Parents also reported that the device appearance was too complex and not “child-friendly” enough, which they believed deterred the child from wearing it.

**Table 7:** Summary of indicators of feasibility for the wearables in everyday life and illustrative quotes from parents

Indicators for Bittium:	Illustrative quotes:
Attaching and detaching	“We felt they were too strongly attached, and it would hurt her.” “It completely fused onto her skin, I couldn’t get it off in the bath. I think if I had to do the pads every day, that would be really hard”
Size and shape	“The clunkiness of it—having to constantly think and not to hold her in certain ways” “Just too big and bulky”
Practicality	“Not robust enough” “I thought, oh my god, this is going to break”
Aesthetics	“It doesn’t look child-friendly” “It wasn’t bright, it didn’t have any characters on or anything like that”
Indicators for NAPPA:	Illustrative quotes:
Ease of use	“I thought it was really quite easy to understand” “The pants were absolutely fine”
Discreet	“It didn’t affect her at all. She didn’t even know they were there”
Labour required	“It was just something else to do”
Practicality	“We did slightly wonder at first how nappy accident-friendly they were going to be”

Compared to the monitor, the NAPPA wearables were reported as easier to use. They were less noticeable to the child, blending into existing routines, and their familiar design enabled discreet implementation, during already occurring changes.

She was absolutely fine, put them on, didn’t notice them, her jamas fit over them and everything, and she just seemed to sleep as normal. *–* P002 parent quote.

No cases of accidental removal were reported for the NAPPA. Parents reported hypothetical concerns for fear of the inbuilt sensor digging in or nappies leaking onto them, but this was not experienced in the study. Across the qualitative data, there were indications that the NAPPA may alter bedtime routines, caregiver workload, or child behaviour. The one family that did not use the NAPPA at all attributed this to parental labour involved, and three cases also described them as an extra step requiring more work at bedtimes that were often already tasking. For example, one parent described the other parent’s unwillingness to put the nappy on.

I didn’t put the kids to bed on the Wednesday night, and [partner’s name] was like, you know, “It’s a bit of a…” well, me and my husband, we both normally put the kids to bed together, so I was just like “We’ll just leave it”, because it’s hard enough, when there’s only the one of us. *–* P003 parent quote.

This additional labour may impact the parent directly, making bedtime routines more challenging or longer, or indirectly, such as by impeding pre-existing sleep routines. For example, in case P004, the child wore the NAPPA overnight but not for daytime naps. The parent described how she values the naps, and thus if her child grew sleepy and she felt the nap was required, she “just puts them in the cot” to sleep. Application of the NAPPA would disrupt this, waking the child, and prompting negative outcomes, reported as inability to complete housework tasks, or the child having insufficient sleep, thus growing upset more easily across the day from tiredness.

She didn’t wear the napping pants for naps, just because… it was something else to do! [looks apologetic] … Before bedtime, we’re getting them changed anyway. So that’s when we put it on. But for naps, I’ll just like… put them in the cot. And not get them changed. – P004 parent quote.

It was not only parents who objected to the additional labour, but the children too. Parent P005 described having to change the nappy unexpectedly when the child was otherwise ready for bed, with the NAPPA applied. This activity had upset the child, and afterwards they refused the NAPPA application the second time. This event was also recorded in the parent diary entry:

17:45 - Refused to wear nappy pants, threw across room. – P005 parent diary entry.

While the need to change triggered this event, the extra “layer” of the NAPPA application caused upset, refusal, and protest. This highlights how the wearable could potentially introduce resistance into routines or moments of care, as well as more broadly in how parents prioritise their child’s comfort and emotion over adherence to wear.

During activity outside of the home, data across the methods showed that wearables use was significantly reduced, particularly if the child was in the care of a different adult than the one in the study, or in daycare. If the study parent was at work, this was correlated with reduced wear, blank pages in the study diary, or significantly shorter entries without time stamps.

One parent kept the wearable on for the nursery, and this was met by excitement and interest, but staff could not reattach the monitor for “health and safety” reasons, and data collection stopped when it fell off. Parents who removed the wearable for daycare attributed this to fear of loss or damage, and not wanting to trouble others with the additional task, worry, or potentially bothersome involvement. For one case, while the child was staying with grandparents overnight, the NAPPA was worn but the diary stated, “Grandma could not get app to work”; thus no data were recorded. Later, the parent described the effort of teaching made difficult without time:

(….) if I’d have had the time to sit down and show her [grandmother], but because… it was the way my schedule was, he [father] was away that weekend, and I was… like it was difficult. I was just trying to explain as quickly as I could. – P005 parent quote.

Overall, across both wearables, other consistent messages were that despite the enthusiasm, best intentions, and effort by parents, the design features of the wearables prevented feasible continuous data collection in everyday life and exacerbated existing parental labour. These problems in workability caused disappointment to parents, as described for example by P003:

I think information, for me, is really important, is really vital. You’re always trying to find patterns and connections. Not just about the sleep but everything... I am pretty sad that it didn’t work... I think I wanted it to work [snaps fingers] instantly, like that, for her to go “Ooh yeah!” and put it on. But in hindsight, of course she wouldn’t want to do it. You know? It was a thing that didn’t look like it was built for children. – P003 parent quote.

This observation demonstrates the potential for wearables to advance understandings for parents, as well as their high potential to build on and make the most of pre-existing interest and motivation.

#### Potential insight from the recorded data

**Fig. 2:**
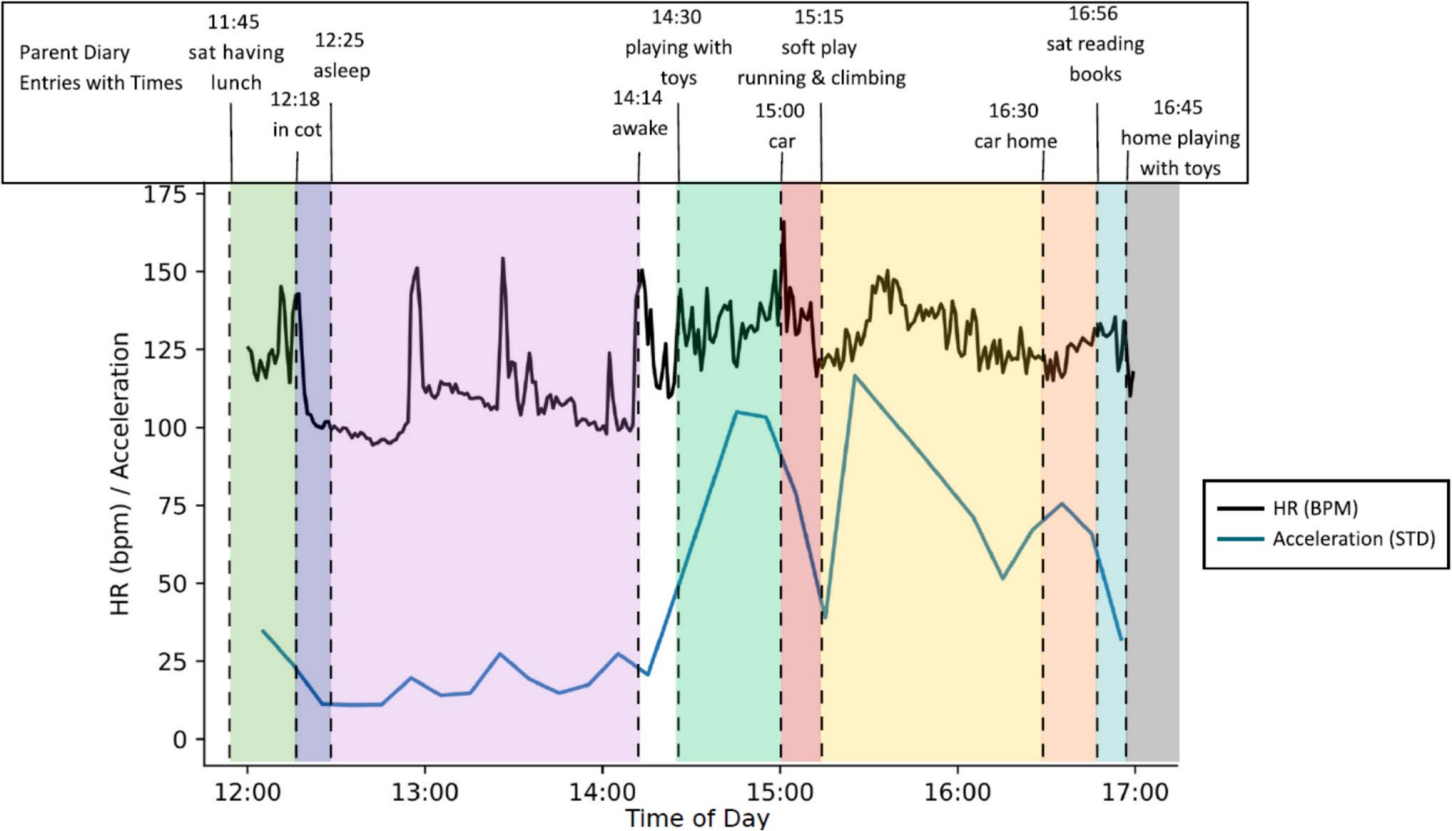
Example timeline mapping physiological data against parent (P002) diary events

We found that it was feasible to combine quantitative data from the devices and the coded behaviour data from parent diaries to create integrated biopsychosocial summaries that overlay moment-by-moment physiological, behavioural, and contextual data. Figure 2 illustrates this in relation to one participant’s data, showing the child’s everyday behaviours as labelled by and recognisable to the parents and the child (e.g. “asleep” and “playing with toys”), together with the child’s continuous heart rate and objectively measured movement (acceleration). This illustrates the potential of the multidimensional, integrated data, both within case descriptions and with a larger data set, for investigating how everyday activity situations relate to the children’s physiological responses and objectively measured behaviour.

**Fig. 3:**
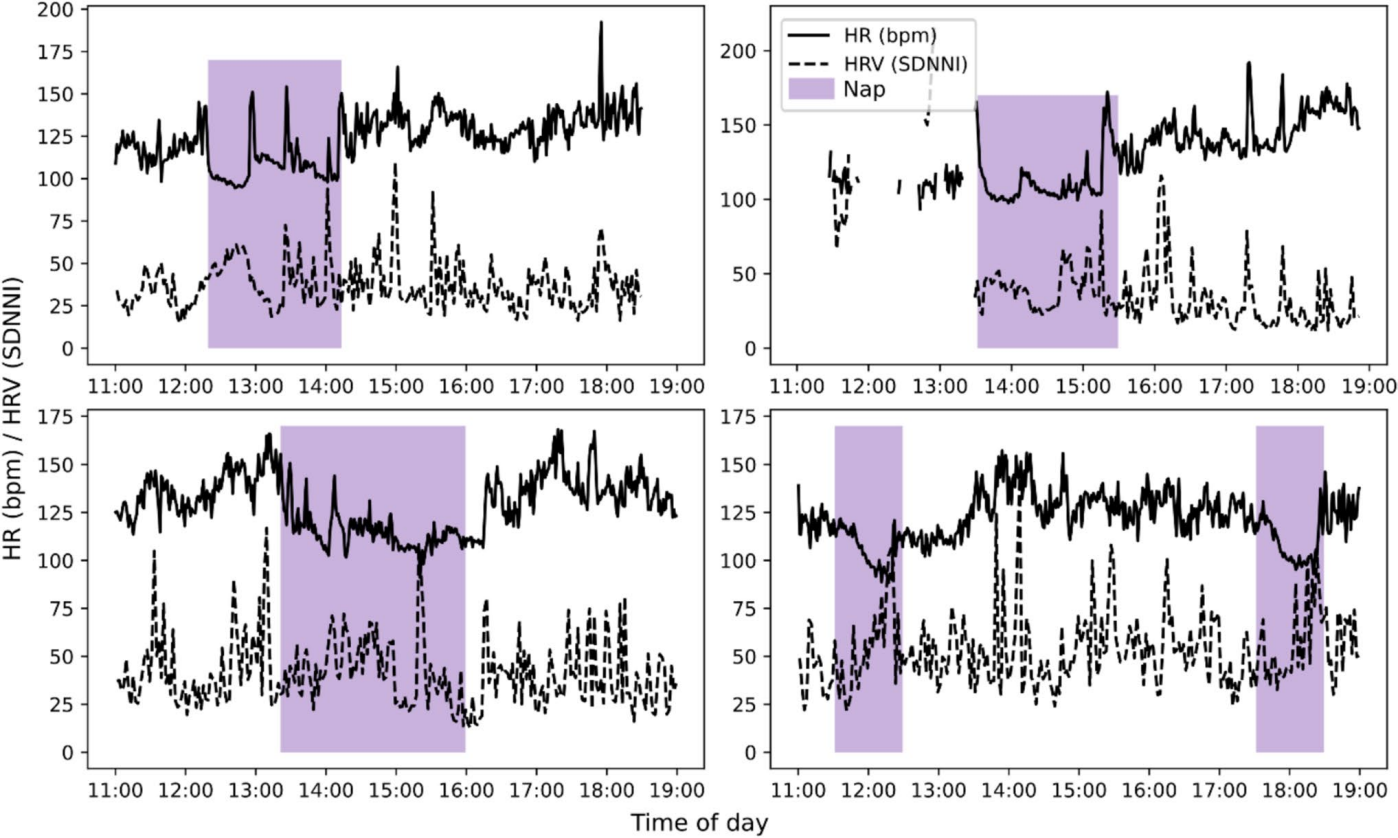
Heart rate (HR, solid lines) and heart rate variability (HRV, dashed lines) during five daytime naps, from two children (top row P002, bottom row P001)

We also confirmed that it was possible to use the data obtained to explore physiological responses to two everyday sleep behaviours: daytime naps and nighttime sleep quality. Figure 3 illustrates the changes in heart rate (HR) and heart rate variability (HRV) captured during naps in two participants. Changes in HR or HRV should be interpreted relative to the baseline for the child, and so we plotted the *z*-score for each, indicating the number of standard deviations from the mean value for each point recorded for that child. In both cases, the HR initially drops below baseline, indicated by the *z*-score dropping below −1 (one standard deviation below the mean). The data show an increase in HRV over 2 SD above the mean in both cases, implying a shift in autonomic balance during the nap.

**Fig. 4:**
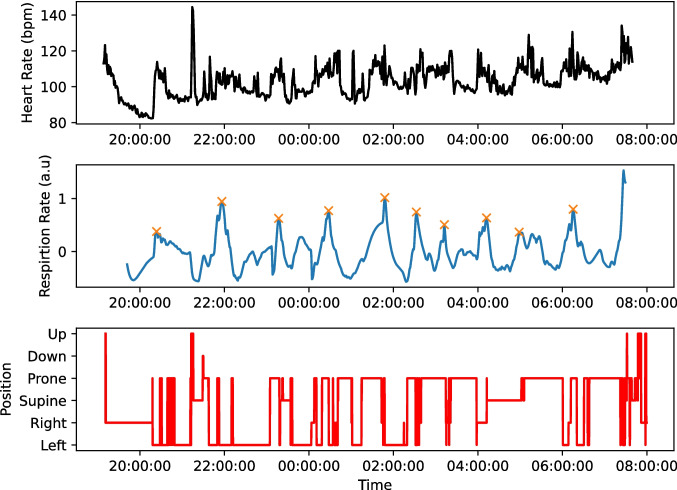
Identifying ultradian cycles and sleeping position using the Bittium 180 heart rate monitor and NAPPA sleep monitor. Top panel shows the heart rate of the child over the night, with a slow rhythm clearly present. Middle panel shows the respiration rate, filtered to isolate the component, with a period between 30 and 120 min. Crosses indicate peaks in the ultradian cycle, with a period of 65 min. Bottom panel shows the sleeping position as detected by the NAPPA sleep monitor

**Fig. 5:**
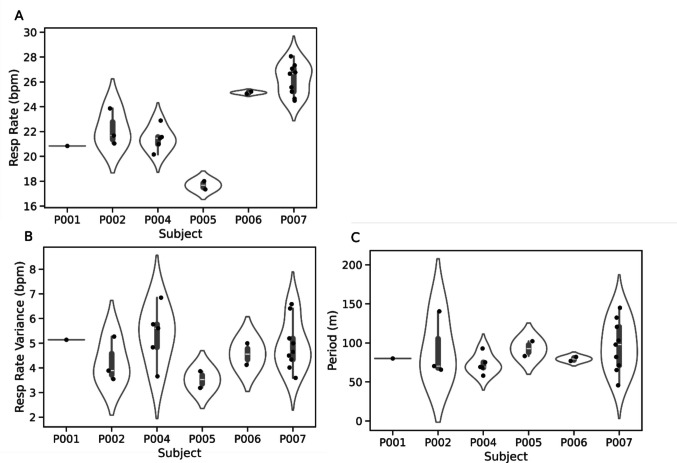
Metrics calculated from the NAPPA for each child who wore it. Each dot represents one night of wear for the child. Left shows the mean respiration rate. Centre shows the variation in respiration rate during each night. Right shows the period of the ultradian cycle in respiration rate for each night

Furthermore, we were also able to detect ultradian sleep cycles in the respiration rate. Figure 4 shows an ultradian rhythm in heart rate recorded by the heart rate monitor, and an ultradian rhythm in respiration rate and sleeping position recorded by the NAPPA. We were able to detect and extract an ultradian cycle in respiration rate from six participants. Figure 5 shows the respiration rate, respiration rate variance, and the period of the ultradian cycles for each night of recording across these six participants.

## Discussion

The present study explored the accuracy and scalability of two wearable technologies for quantifying young children’s everyday sleep and physical activity over several days, in a physiologically informed manner. We found that family initial impressions were positive, with enthusiasm for trying out the wearables; however, as the study progressed into the reality of using the wearables at home, issues emerged. The heart rate monitor was found to be often unworkable for children and parents in everyday life, showing that it would not be suitable for scaling up at the population level. The NAPPA was experienced more positively, with higher wear time and better workability into routines due to its familiar, discreet appearance.

In terms of limitations, the main impedance to generalisability is the small sample size, which reflects the study’s exploratory nature as well as parents’ concerns about usability. The pool of invited participants was large, estimated around 2,500, and the limited number of responses and actual study completions was a key part of answering the study question, suggesting that delivering the present study protocol at the population level would likely be very challenging. The small sample size also limited generalisability in terms of the diversity of the participants, especially in relation to socioeconomic background and geographical location. Because of the usability challenges, the resulting physiological data were limited, with related limitations to external validity; thus, no generalisable conclusions about child physiology should be drawn from the present data. A further limitation was the length of time to trial the wearables; more comprehensive results could be obtained with longer than a single week of use.

Strengths include the complementarity of methods. The use of an ethnographic investigation with families alongside quantitative wearable data provided a nuanced understanding of the everyday factors affecting the feasibility of wearables use, including real-life challenges—those that are related to lifestyle, variance in everyday activity, and social attitudes—that may otherwise be unreported. Additionally, integrating play into the study protocol allowed for playful exploration and increased interest in the wearables for the children and parents, which highlights the potential for play as a tool for wearable use or adherence in child studies. Collecting real-time, all-day data on infant behaviour is unprecedented, opening new avenues for understanding infants' everyday experiences (Franchak et al., 2024).

The present study is, to our knowledge, the first to collect multidimensional, parallel behavioural and physiological data on very young children’s everyday health behaviours in natural settings, longitudinally over several days. The results indicate that using wearable devices can bring subtle shifts in both parent and child behaviour, for example by disrupting routines, as well as shaping parent–child dynamics, e.g. by frustrating or worrying the child. However, these results require further investigation in larger studies with wearables that are more usable and acceptable to parents. The present study provides new insights that can inform the design of such devices for young children for use in the everyday population health contexts, instead of the current clinical, shorter-term measurement for diagnostic purposes (Memon et al., 2019). The present study also complements the existing literature on the experiences about wearables (McElwain et al., 2024) in older children (Oygür et al., 2021; Mackintosh et al., 2019), providing insights that are specific to young children, currently an overlooked population (Cibrian et al., 2021). Specifically, the findings can be used to inform the development of both new devices and analytical methods for integrating other sources of data with these data. The integration of parent-diary data with physiological and objective behaviour measures illustrates further opportunities for future studies—on one hand, for studies seeking to advance a more granular understanding of biopsychosocial mechanisms underpinning early-life health and behaviour, and on the other hand, for intervention development studies that seek to promote early-life health, sleep, or physical activity through HealthTech.

While physical activity and sleep measurement in children under 3 is currently underserved, the studies in clinical populations have begun to develop sensors and methodologies with potential for further scaling. Our findings corroborate quantitative evidence demonstrating the NAPPA to be feasible for out-of-lab studies (de Sena et al., 2024), and provide a complementary, independent qualitative assessment. A combined infant remote monitoring system for movement, pulse rate, and respiration rate—aiming to measure sleep and physical activity—is also currently being trialled, with preliminary data now available (Pini et al., 2024). Part of the HEALthy Brain and Child Development Study (Nelson et al., 2024), this utilises a watch-like wearable photoplethysmography (PPG) sensor to capture the pulse rate of the child. Our work provides important qualitative insights into how these new technologies will impact the families asked to use them.

Overall, the long-term adoption of wearable technologies and their associated challenges remain a widespread issue to be addressed (Ates et al., 2022). Our study involved spending time with children and parents, facilitating an understanding of user experience, including both essential positive and negative experiences (Vermeeren et al., 2010) with the child, convenience, and experience at the centre. The generation of parents and children in our study are predicted to drive demand for wearable technology (Chotiyaputta & Shin, 2022), and their input is integral to the process (Steen, 2013).

In conclusion, there is enthusiasm and potential for the use of wearables with young children to develop deeper and richer understandings of the physiology of their everyday behaviours at home. This study demonstrates promise for wearables in offering insights into the physiology of everyday events, such as heart rate during daytime naps and detecting ultradian rhythms in nighttime sleep. However, current wearables have usability challenges that hinder their scaling up for larger longitudinal population health studies. Co-creation of child-friendly, practical hardware and software is therefore essential for effective, large-scale health monitoring in young children.

## Data Availability

The quantitative data and analysis data are available (see below for repository links). Due to the sensitive nature of the qualitative data (e.g. ethnographic transcripts, audio), these data are not openly available. A summary of the themes and findings from the qualitative analysis can be accessed in the manuscript—if you would like to discuss access to the original qualitative data, please contact the corresponding author. The experiments were not formally preregistered, but their full description was included in the protocol as part of the independent ethics submission. The research protocol is openly available here: https://data.ncl.ac.uk/articles/report/WearAble_Project_Protocol/21608838?file=38463158. The supplementary files for this manuscript are available here: https://figshare.com/s/c3336a1a329a75dd67a1.

## Code availability

Code used to produce the figure and analyses presented is available here: https://github.com/haeste/Child_Wearables_Pilot/
